# What Happens Before Book Reading Starts? an Analysis of Teacher–Child Behaviours With Print and Digital Books

**DOI:** 10.3389/fpsyg.2020.570652

**Published:** 2020-11-12

**Authors:** Trude Hoel, Elisabeth Brekke Stangeland, Katrin Schulz-Heidorf

**Affiliations:** ^1^Norwegian Reading Centre, University of Stavanger, Stavanger, Norway; ^2^Faculty for Educational Science, University of Hamburg, Hamburg, Germany

**Keywords:** picture book apps, print picture books, joint attention, prior knowledge activation, early childhood education and care, shared reading, paratext

## Abstract

A body of research documents teacher–child reading behaviors in educational settings. Few will disagree that the potential for word and narrative comprehension increases when children’s prior knowledge is activated and when children’s focus is fully on the reading session. Despite this, little is known about the potential for establishment of joint attention and activation of prior knowledge in an early childhood education and care setting and how early childhood educators prepare young children to participate in shared book reading sessions before formal reading starts. Based on video data of teachers (*N* = 12) and small groups of children (*N* = 72) reading picture books and picture book apps in kindergarten, we sought to shed light on what behaviors occur *before* reading starts. The analyses were conducted in two phases. The first phase was based on 48 videotaped readings and followed a descriptive quantitative approach to investigate early childhood teachers’ time use before the reading session, with readings of both print books and picture book apps. The second phase was based on two app readings in which the pre-reading phases stood out for their long duration. A qualitative analytical approach was applied to describe the teacher–child behavior, establishment of joint attention, and activation of prior knowledge during the two specific pre-reading events. Even though the sample is small, we find clear examples of pre-reading strategies specific to app readings. In this study, we discuss teachers’ strategies to promote joint attention and activation of prior knowledge in new ways and how teachers exploit the pre-reading phase, for instance taking advantage of the books paratext, while adapting to the medium. Nevertheless, there remains a knowledge gap concerning app readings with short or no pre-reading phases.

## Introduction

Shared reading with young children in early childhood education and care (ECEC) settings^1^ involves children and teachers directing their attention toward the forthcoming activity and the book to be shared. Shared reading is in this study understood as verbal alternations between the mediator of the story and the children in order to encourage the children to participate in extended discourses, contributing to the children’s early literacy and language development (Dickinson and Morse, 2019). The pre-reading time period offers valuable opportunities to support children’s comprehension during the shared reading sessions. Activating children’s prior knowledge and helping them connect their reading to their background knowledge (Keene and Zimmermann, 1997) is an effective way to improve children’s understanding of the text being read. The parallel processes of having to create a mental representation of information contained in the text and decoding the language units might constitute a cognitive load (Bishop, 2014). Given that activation of prior knowledge reduces some of the cognitive load on the working memory for storyline processing and even language learning, the pre-reading phase is of great significance from a language stimulation perspective.

Further, joint attention, where children’s and teachers’ mutual engagement is coordinated with their common focus on the book (Tomasello et al., 2005), can serve as a scaffold for children’s engagement in the reading activity. Communication and coordination in preparing and participating in shared efforts involves making adjustments among the participants, “stretching their common understanding to fit new perspectives in the shared endeavor” (Rogoff, 1997, p. 272). Establishing a frame of joint attention from the start can help teachers re-establish joint attention and draw the children back to the story when they become distracted during the shared reading.

In this way, a teacher’s task prior to the reading session is to create engagement for both participation in the reading activity and for the story itself. Despite this understanding of the key roles of joint attention and prior knowledge, very few studies have explored what happens *before* the reading with groups of children in ECEC settings begins. In this regard, it is of interest to determine whether kindergarten teachers spend time on pre-reading activities at all. In addition, if they do spend time on pre-reading activities, what do they actually do within this time period?

While print books are traditionally used in shared reading, the digital medium is becoming increasingly applicable. A body of research has shown that shared reading of both print picture books and picture book apps enhances comprehension (van den Broek et al., 2017) and vocabulary growth (Mol et al., 2008, 2009; Mol and Bus, 2011). This positive effect can be attributed to the talk that surrounds the storybook reading (Pesco and Gagné, 2017). The effects of the content of teachers’ talk and what pre-reading strategies teachers use have yet to be explored.

In this study, we are interested in what happens before shared reading starts, and our main research questions are as follows:

– How much time is spent on establishing joint attention and activating prior knowledge before reading print picture books and picture book apps?
– How does establishing joint attention and activating prior knowledge manifest in two examples of app readings characterized by the longest pre-reading phase durations in the sample?

The present study contributes to the field in three main ways. First, by using video observations of ECEC reading sessions of print picture books and picture book apps, we illuminate whether kindergarten teachers promote joint attention and activate prior knowledge before shared reading with groups of 4- and 5-year-olds. Second, by exploring what actually happens before reading starts in two selected reading events, this study gives special consideration to the practical implications of activating children’s prior knowledge and establishing a frame of joint attention. Third, the present study fills the gap in the understanding of adult–child behaviors before a shared reading session. With this triple focus, we want to ascertain the possibilities and limitations of print picture books and picture book apps, which could contribute to understanding of the qualitative markers of optimal design for children’s print and digital books.

## Study Background

Prior to shared reading, teachers have the opportunity to provide texts and media in ways that promote and secure children’s participation in the session. In a typical Norwegian ECEC setting, before the reading session, children and teachers come together to calm down and focus on the book. They may explore the front and back cover, discuss details of the illustrations or what the title of the book might mean, or if they have read the book earlier, retell parts of the story for each other (Hoel et al., 2011). In this vein, the study background is based on two well-established psychological variables, namely, *joint attention* and *prior knowledge*. In addition, the study draws on literary studies that do not empirically investigate children’s engagement with books but describe in detail the relevant or accessible parts of the book for pre-reading conversations and explorations, that is, the paratext.

### Joint Attention

When children and teachers share their associations and reflections surrounding the story to be read, they pay attention to each other and to the utterances related to the book. Tomasello (2003) refers to this coordination of attention to each other and a third event (or object) as *joint attention*. Joint attention was found to correlate with children’s word learning and is therefore considered to play a critical role in vocabulary development (Carpenter et al., 1998). However, in the context of shared reading, establishing a frame of joint attention prior to the reading activity can also play a crucial role in maintaining children’s attention and can, through this already established engagement, help them come back to the story when the reading is disrupted. In this way, establishing a frame of engagement and joint attention serve as a scaffold for children’s attention and therefore their story comprehension.

A prominent part of the teacher’s scaffolding (Wood et al., 1976) is thus to encourage joint attention among learners. This scaffolding strategy, known as *recruitment*, revolves around getting children interested and engaged in the learning activity and creating a common ground for shared reading. The teacher may contribute to the children’s expectations of the joint activity by expressing his or her own expectations. Another pre-reading scaffolding strategy is to refine the children’s area of attention, described as a *reduction in degrees of freedom* (Wood et al., 1976). With the reduction in degrees of freedom technique, teachers design a reading situation that allows the children to be active participants while the teacher stays in control, for instance, by removing potential distractors. The teacher can ensure that all children are able to see the book or the screen well, that toys are cleared away, and that inappropriate behaviors do not disrupt the session. This scaffolding strategy must also be understood in relation to the medium one reads from, as reading from a touch screen in an early education setting is different from reading print books (Hoel and Tønnessen, 2019). Reading from a touch screen in kindergarten also differs from screen reading practices established in homes, where reading in dyads is the most common practice (Tønnessen, 2016).

### Prior Knowledge

According to Rogoff (1997, p. 272), individuals transform their understanding of and responsibility for activities, such as shared reading, through participation, where they make contributions either in actions or in “stretching to understand the actions or ideas of others.” To contribute to the children’s comprehension of the text, the teacher can help them activate prior knowledge. Prior knowledge involves insights that the individual children already have when they encounter a text. Within reading research, the importance of utilizing readers’ prior knowledge and experiences, also called pre-understanding, is well documented (Bråten, 2007; Roe, 2008). With prior knowledge, the children can connect the new and unfamiliar elements that they encounter in the text with what they already know. In communicating these insights before reading starts, all participants stretch their understanding, thus increasing their understanding and expectations of a text before it is read.

Children’s prior knowledge can be prompted with questions, which are widely used in interactions between educators and children. In her research in Norwegian kindergartens, Bae (2004) distinguishes between closed questions, which signal the expectation of a given answer, and open questions, where the children can answer more freely. Questioning statements of the open type are equivalent to appreciative communication, which invites reflection and common wonder, while closed questions are assumed to undermine independent reflection (Bae, 2004, p. 87). Questions such as “What do you see in the picture?,” “Does this remind you of anything?,” “What can this title mean?,” and “What do you think this book is about?” may be used to retrieve the children’s prior knowledge. In parallel, prompting questions might enhance children’s interest in the book as well as their expectations for the activity, which are vital parts of the actual reading experience.

### The Role of Paratext

The term “paratext” describes all textual elements that come with a text and that influence how the text is interpreted. Examples of such textual elements may be the front page and colophon. Before reading starts, readers will relate to a book’s paratext to a greater or lesser extent (Genette, 1997). The central text is surrounded by other textual resources, operating at different levels, in both external and proximate relation to the main text. The paratext acts as a link between the institutional framework of the text and the text itself. At the same time, the paratext serves as a series of entries that the reader can use to establish joint attention and prior knowledge before the reading starts and to interpret the text. The paratext has verbal and visual functions; it prepares, presents, and contains information about the content of the book. The paratext thereby forms part of the book’s meaning potential and can condition and even change the reader’s reception of a text.

Genette (1997) further distinguishes between peritext and epitext, where the former involves the textual elements that have a clear connection to the main text without being an explicit part of it and the latter refers to those parts of the paratext that are clearly separated from the main text itself; thus, the paratext can either be an addition to the story or an integrated part of the story.

Opening a picture book app may have some similarities, yet some features differ from those of a print book. Picture book apps are available within digital media; therefore, the first step is to turn on the tablet and choose the right app. In some apps, the opening page mimics the front cover of the print book, with the title, name of author/illustrator, and illustration; some apps add sound (i.e., an opening tune); and some add movement or interactivity. The app’s paratext also includes menus with options and information for the book app, and in some apps, the opening page is an introduction to how to turn pages, turn the sound on and off, etc.

Zhao and Unsworth (2017) see the app as a semiotic artifact, drawing a distinction between the technological features that form an integral part of the text’s meaning-making (intratextual interactivity) and those that help make the app work but that are not part of the narrative’s meaning-making, such as peritexts and navigation menus (extratextual interactivity). With print picture books, the children’s meaning-making springs from verbal text and illustrations together. With picture book apps, other semiotic resources, such as sound and animations, may also be subject to exploration, display esthetic qualities (Schwebs, 2014), and add to prior knowledge and rich dialogs.

In this study, we are interested in what happens before shared reading starts, and our main research questions are as follows: How much time is spent on establishing joint attention and activating prior knowledge before shared reading of print picture books and picture book apps? How does establishing joint attention and activating prior knowledge manifest in two specific examples of app readings characterized by the longest pre-reading phase durations in the sample? Based on empirical material with videos of teachers and small groups of children reading print picture books and picture book apps in ECEC settings, we aimed to ascertain what happens prior to the start of a reading session.

## Materials and Methods

Data from the project “Books and apps: Developing an evaluation tool for e-books targeted towards children” (VEBB) (Mangen et al., 2019) were used. In the overall project, the teacher’s educational aim is to facilitate language learning by providing challenging content that requires the use of language to be explored and shared (Grøver, 2018), and both print picture books and picture book apps are the basis for dialog-based shared reading (Mol et al., 2008; Burger, 2015).

### Participants

The VEBB project involved 12 kindergarten teachers in six kindergartens. All 12 kindergarten teachers have a bachelor’s degree in early childhood education. Each teacher carried out four reading sessions, reading two titles in both the print book version and the app version with the same groups of up to six children (*M* = 57.2 months, SD = 7.9), for a total of 72 children.

The study was approved by *The Norwegian Social Science Data Service*, a third-party ethical supervisory agency in Norway. All participants were informed about voluntary participation and the opportunity to withdraw during the study. Each child’s parents gave written informed consent for their child to participate. In addition, the children gave verbal informed consent to participate. Neither the children nor the kindergarten teachers can be identified in our work. To preserve the children’s anonymity in the videotapes and still allow for their recognition between films, they were given number tags ranging from 1 to 6 to place on their jumpers.

### Preparing and Videotaping Reading Events

In preparation for the study, the teachers participated in a workshop on shared dialog-based reading of print and on-screen books. In this workshop joint attention, prior knowledge and the function of books paratext were highlighted. Following this, for the next 6 months, they had access to both types of media for practice.

The teachers were in charge of putting together reading groups that take into account the children’s interests, mastery of language, and group interaction, as well as the physical conditions to optimize the children’s engagement and participation (Hoel and Tønnessen, 2019). The teachers were free to design and conduct each reading. Children who were not present on the day of the filmed reading were not replaced. The reading events were videotaped by the kindergarten teachers themselves over the course of 3 weeks. In the videotaped readings, each teacher read two titles in their print and app versions, making the total number of filmed sessions 48. The reading order of the print and app versions was reversed for the readings of the second title to secure balance in the overall design.

### Coding the Videos

The video data were entered into the INTERACT video analysis program (Mangold, 2010, Lab Suite Version, Program Version 16.4.0.56), a video coding and analysis software for observational studies that can be used as an interface for both quantitative and qualitative coding. A coding scheme was developed, adapting categories from previous studies of children’s engagement with picture book apps (Roskos et al., 2012; Merchant, 2015) and adjusting them to cater to the focus on the purpose of the study. Four coders took part in the coding. Intercoder agreement was checked, with four films being independently coded by two coders each, and intercoder reliability was found to be acceptable: kappa reached a level of *κ* = 0.71 for the frequency codes and *κ* = 0.60 for the duration codes (Mangen et al., 2019).

There are two main categories of codes: duration codes, which record how long a phenomenon lasts, and frequency codes, which record the number of instances. All frequency codes are linked to either the children or the teacher to identify the active party. For the quantitative analysis in this article, the applicable code is the duration code labeled *Pre-understanding*, characterized in the coding scheme as the “time spent on establishing shared focus before the reading starts: concerning expectations and background knowledge related to the story, the medium and/or the participation” (Mangen et al., 2019, p. 94). For the qualitative analysis in this article, video recordings of the specific time interval prior to the reading activity (coinciding with the duration code) were transcribed using the CHAT (Codes for the Human Analysis of Transcripts) standardized transcription system (MacWhinney Brian, 1991) for a selection of videos. For the purposes of this article, parts of these transcripts have also been translated from Norwegian to English.

### From Quantitative to Qualitative Analysis

The analysis was conducted in two phases. In the first phase, we used qualitative coding of video data in quantitative analyses to reveal how much time the kindergarten teachers actually spent on establishing joint attention and activating prior knowledge before the reading started. The data were imported into SPSS 21 for descriptive frequency analyses. The duration time in each film was analyzed, both in print picture books and in picture book apps. Based on the descriptive analyses, two reading sessions stood out for their long duration (3 min 6 s and 3 min 7 s), and these sessions were selected for further inspection in the second phase.

The second phase was an exploration of what happened before the reading session in these two specific picture book app reading events. By analyzing teacher–child pre-reading behavior for the two specific sessions, we identified strategies for establishing joint attention and prior knowledge in picture book app readings. The distinctive strategies were formulated (Table 1) both theoretically, based on the literature (Cresswell, 2007) and based on the data through a combination of inductive and deductive analytic approaches (Riessman, 2008).

**TABLE 1:** Strategies for joint attention and prior knowledge.

**Pre-reading**	**Strategies**	**Description**
Joint attention	Recruitment	Engaging; expressions of curiosity; humor
	Maintaining attention	Elaboration; questions; corrections
	Reduction in freedom	Organization; corrections; rules
	Retrieval of attention	Questions; distractions; touching
Prior knowledge	Open and closed questions	On the literary elements; on the medium; on reading experiences
	Confirmation	Of the children’s associations

In the result and discussion part, we give brief descriptions of the two selected reading events as well as rich descriptions of the paratext of the two picture book apps. We present the results continuously in the discussion and use excerpts from the transcripts to exemplify.

### The Story Titles

The following four titles, all available in both print and app format, were selected for use in the video observation study: Jansson (1952/2017), *The Book about Moomin, Mymble and Little My [Hvordan gikk det?]* (available in English); Stai (2008), *Yesper and Noper [Jakob og Neikob]* (available in English); Aisato (2014), *A Fish for Luna [En fisk til Luna]* (not available in English); and Charlotte Bråthen and Markhus (2013): *The Seed [Frøet]* (not available in English). The selection of the titles was based on the following selection criteria: the titles are available in Norwegian, in print and app format; there is ample potential in the story and the text for rich dialogs; the apps display a variety of interactive options; the books and apps are of high linguistic and esthetic quality; the theme/topic/content in the stories is relevant for both boys and girls and age-appropriate (age 4–5 years); and the books and apps display a diverse verbal language from simple words and sentences to more complex language in which the wording might generate curiosity and invite readers to explore vocabulary, metaphors, etc. (Mangen et al., 2019).

## Results and Discussion

In the following, we will present our analysis and continuously follow up with discussions. We will start by addressing the quantitative material in order to answer the first research question: How much time is spent on establishing joint attention and activating prior knowledge before reading print picture books and picture book apps?

**FIGURE 1:**
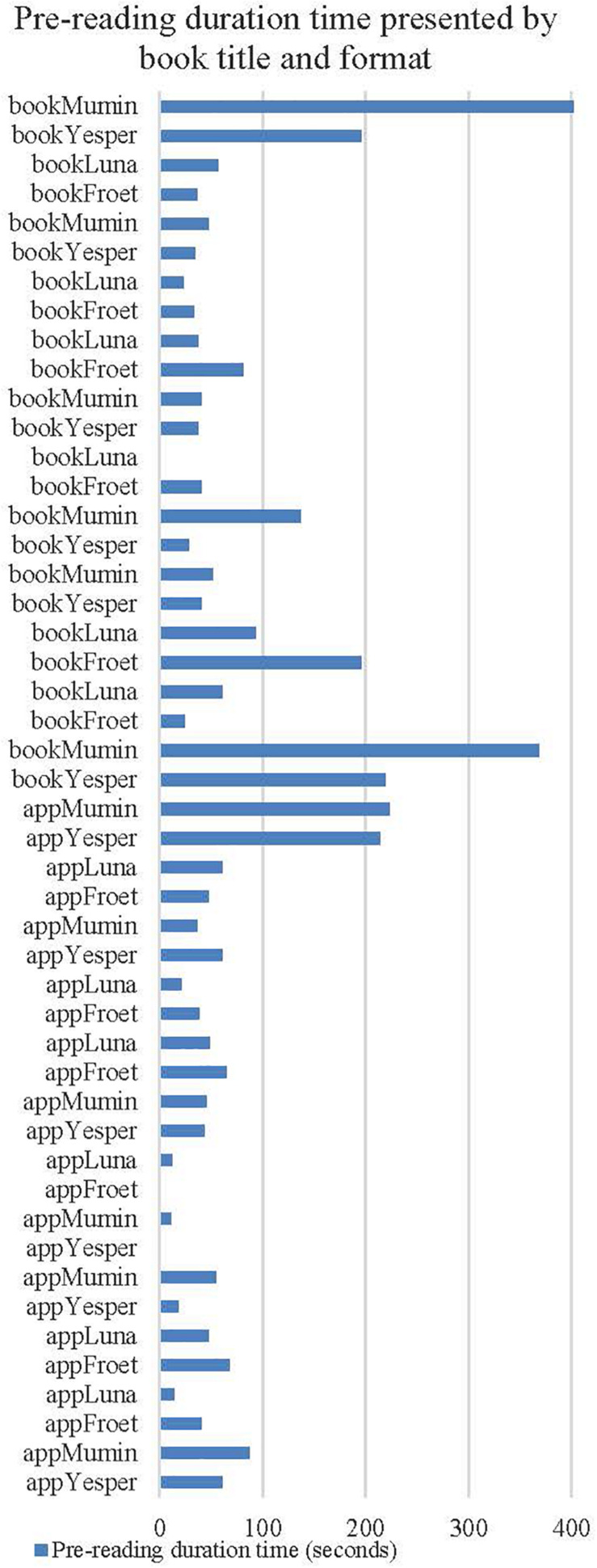
Pre-reading duration time in each reading session presented by book format and title.

Table 2 presents the distribution of the pre-reading time in all reading sessions in our material. Based on all the videotaped reading sessions, the frequency analysis shows that on average, the pre-reading phase represents 5.4% of each reading session (*M* = 76 s, SD = 89.84). As expressed by the standard deviation, the pre-reading time varies greatly between each reading session. The longest sequences are found for the print book readings (Figure 1). For example, in one reading of the print version of *The Book about Moomin, Mymble and Little My* (Jansson, 2017), the pre-reading time is 7.5 min. In other reading sessions, the teacher starts the reading immediately, without any pre-reading phase.

**TABLE 2:** Pre-reading phase duration time for digital and print picture books.

	**Reading session**	**Pre-reading phase**		
	**Total duration time (mean duration time)**	**Total duration time (mean duration time)**	**Mean duration time in %**	**SD**
All reading sessions (48)	18 h 47 min (*M* = 39 min)	1 h 25 s (*M* = 1 min 16 s)	5.4%	89.84
App readings (24)	9 h 6 min 25 s (*M* = 22 min 46 s)	53 min 35 s (*M* = 55 s)	4.0%	55.24
Print readings (24)	9 h 40 min 30 s (*M* = 24 min 11 s)	38 min 35 s (*M* = 1 min 36 s)	6.6%	111.92

The results also show that the absence or very short duration of the pre-reading phase was slightly more frequent for the app readings than for the print book readings (see Figure 1). In addition to the teachers’ pre-reading strategies, this could point to that the medium—print or digital—might have influence on how much time is spent on establishing joint attention and activating prior knowledge before reading starts. However, this should be tested in a bigger sample.

When the medium is a picture book app, on average, 4% of the whole reading session is spent on pre-reading (Table 2). When the medium is a print picture book, 6.6% is spent on pre-reading. However, the variation in pre-reading time during the app readings (SD = 55.24) is noticeably smaller than that during the print book readings (SD = 111.92). This might point to that the digital medium imposes certain frames that make the teachers’ strategies for establishing joint attention and activate prior knowledge relatively homogeneous. Does this imply that teachers struggle with establishing joint attention and prior knowledge when preparing to read the picture book app, or does the digital book, with its medium specific epi- and peritextual features, provide poor conditions for establishing joint attention and prior knowledge? This leads us to phase 2 of the analyses. Inspired by the results showing that the pre-reading phase in app readings is relatively short, we explored to a greater extent what happens before reading using the recordings of the two longest app readings in the sample.

In phase 2, we explore what happened before reading started in two specific app reading events, and the research question is as follows: How does establishing joint attention and activating prior knowledge manifest in two examples of app readings characterized by the longest pre-reading phase durations in the sample?

Going back to Figure 1, two of the app readings stand out in regard to the duration of the pre-reading phase; a longer duration theoretically presents a greater opportunity than a shorter duration of this phase for pre-reading activities. The picture book app *The Book about Moomin, Mymble and Little My* (Jansson, 2017) was read in one of these sessions. *Yesper and Noper* (Stai, 2008) was read in the other. One is a first reading and the other a second reading, and the teacher is the same in both readings.

### Before Reading the Picture Book App *The Book About Moomin, Mymble and Little My*

In video observation 612appMU1, a kindergarten teacher and six children (two boys and four girls) prepare to read the picture book app *The Book about Moomin, Mymble and Little My* (Jansson, 2017). The children sit on low chairs in a semicircle, and the teacher sits in front of them on a stool with wheels. On their sweaters, the children have stickers with numbers from 1 to 6. The teacher holds the tablet facing the children. She stretches it forward when they ask to see and to study the details in the illustrations, and she gives them the opportunity to tap/touch the screen. The teacher’s strategy, the “show strategy” (Hoel and Tønnessen, 2019), is characterized by the teacher facing the children—not the screen—so that she meets the children’s gaze, sees where they are turning their attention, and controls the children’s access to the screen. The children have read neither the print nor the app version of the book earlier, although the classic Moomin universe may be familiar to some of them.

### Paratext in the Picture Book App *The Book About Moomin, Mymble and Little My*

**FIGURE 2:**
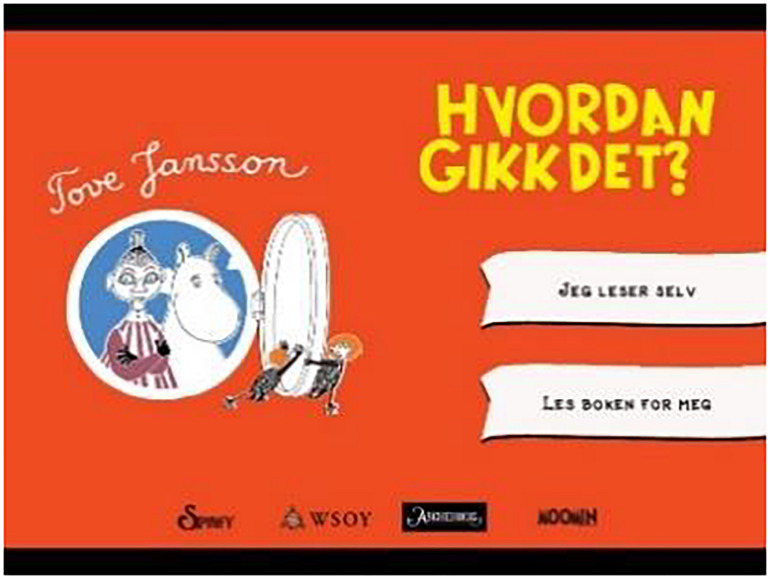
Opening page of the app *The Book about Moomin, Mymble and Little My.* Screenshot reproduced with permission from Aschehoug and Rights and brands.

The opening page in the app displays the name of the author, book title, and manufacturers (Figure 2). There is an illustration of two trolls opening a lid on the left side of the screen. Below the lid, two of the book’s characters, Mymble and Moomin, are portrayed. When the page is open, there are ongoing bird chirps. The page contains two banners on the right side, where the readers can choose between “Read by myself” and “Read to me.” After the reader has chosen, page two appears.

**FIGURE 3:**
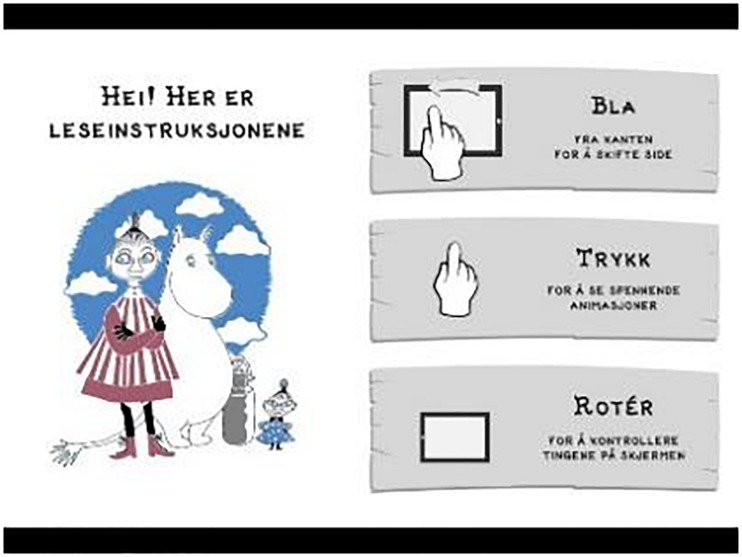
Second page of the app *The Book about Moomin, Mymble and Little My.* Screenshot reproduced with permission from Aschehoug and Rights and brands.

On page 2 (Figure 3), the readers are addressed with the text: “Hi! Here are the reading instructions,” and below the written text, there is a full body illustration of the main characters Mymble, Moomin, and Little My. On the right side of the screen, there are three instructions presented as written text and small animations of a hand that performs a movement: “Turn page from the edge to change sides,” “Press to see exciting animations,” and “Rotate to control the objects on the screen.”

In the analysis, we looked at the dialog and interaction between the teacher, the children, and the medium that takes place in the minutes from the teacher turns on the camera until the story starts, after approximately 4 min.

### Establishing Joint Attention

To get the children interested in the shared reading activity, the teacher starts by creating a common starting point before the app is opened. While the children are studying the digital bookshelf on the tablet, where they can see thumbnail images of the different available picture book apps, the teacher encourages them to focus their attention in search of a specific book: “Let’s find a book that we have not read earlier.” Thus, the teacher is recruiting (Wood et al., 1976) the children to take part in the shared search activity. She also asks questions to engage the children urging them to study the front cover illustrations:

T: Which one of these is Moomin?
C: [points]
C: The white one.
T: Do you know who this is? [points]
C: It’s the girlfriend.
T: Are they boyfriend and girlfriend, do you think?
C: Yes/No.
T: Yes, they might be, I don’t know.

Even though the questions are formulated in a closed way (Bae, 2004) (“Which one of these is Moomin?,” “Do you know who this is?”), they have a practical function in helping the children attune their attention to the story that they are about to read. The questions also create engagement inasmuch as the children actively engage with the paratext both by answering questions and tapping the screen.

This short sequence helps build expectations of who and what the story might be about: Are Mymble and Moomin girlfriend and boyfriend? The teacher, who has prepared for the shared reading and knows the book well, lets the children explore and establish their own prior knowledge, thus establishing joint attention in the whole group.

During the minutes prior to the reading activity, one of the participants in the group applies the strategy of “refining the area of attention” (Wood et al., 1976), and this is not the teacher. The child with sticker number 1 on her sweater finds an opportunity to promote her candidacy to be the first to tap the screen.

C: Can we do it like this: That number one [points to the sticker on her jumper] can turn the page first, and then the one with number two can do it afterwards and then … [goes on to number 6]
T: So we do it one at a time?
T: Is it OK if I decide who will turn the pages?

The organization of taking turns has been highlighted by the introduction of various forms of technology in kindergartens (Arnott, 2018). To control the activity, the teacher decides who is allowed to press hot spots and when, thus reducing the children’s degrees of freedom (Wood et al., 1976). Kindergarten teachers find that this regulation contributes to joint attention on the story and that it makes it easier for the children to follow the narrative (Hoel and Jernes, 2020). However, in this situation, it is not the teacher who seeks to take control of the situation but one of the children. The teacher confirms the child’s input (“one at a time”) before asking the children if it is okay that she, the teacher, be the one who decides.

Rather than using a reduction in degrees of freedom to establish joint attention, the teacher accentuates new interactive affordances of the picture book app presented as part of the extratextual interactivity (Zhao and Unsworth, 2017) to refine the area of attention (Wood et al., 1976).

T: Here it says, “Rotate to control elements on the screen”.
T: That means that we can do like this with it [demonstrates rotation with the tablet].
T: That will be kind of fun.
C: Yes.

By announcing her own expectations (“fun”), the teacher contributes to building the children’s positive expectations of interactive opportunities, which is a fundamental part of establishing joint attention toward the picture book app.

Interestingly, the pre-reading phase in this example starts on the tablet book shelf, at a moment when the book is just a small picture among others. The kindergarten teacher involves the children in searching for the book and, in doing so, takes advantage of the unique potential and features of the app medium. With this, the teacher initiates a frame of joint attention. This teacher is not steamrollered by the medium; on the contrary, she insists on implementing pre-reading, even though the paratext of this specific app does not invite to traditional pre-reading activities.

### Activating Prior Knowledge

During the 4 min before reading starts, the teacher contributes to the children’s comprehension of the text by helping them activate their prior knowledge. With prior knowledge, the children can connect the new and unfamiliar elements that they encounter in the text with what they already know. Even though the picture book app is new to the children, the teacher tries to link the book to children’s assumed experiences with the classic Moomin universe.

T: Do you know who Moomin is?
C: No.
C: I’ve seen her on TV.
T: Can you see Moomin anywhere? [stretches forward and shows tablet]
C: It’s her with the black hair.
T: Can you tap on Moomin?
V: [The child taps at the app. The character is not Moomin.]

The teacher also activates the children’s prior knowledge on how to read a picture book app using the menu in the apps’ paratext.

T: When we turn pages in this book we do like this [demonstrates movement with hand].
T: And then it says “Tap to see exciting animations”.
T: Does anyone know what an animation is?
C: Yes.
T: What is an animation?
C: [hesitates]
T: It’s movements in a way.
C: Yes, like this? [stands up and wiggles].
T: Do you remember the other books we have read, the ones where we sometimes can tap. Like in the Billy Goats. What happens when we tap on the goat’s butt?
C: Like this [stands up and pretends to flatulate, laughs].
T: That is a kind of animation.
T: In this book, there are also animations that we can tap. But I will tell you when you can tap.

The children are experienced readers of picture book apps with interactive features. By activating children’s prior knowledge of the medium, the teacher also establishes an arena for using terminology related to digitization (“animation”), thus contributing to expanding the children’s vocabulary within a frame of shared attention where the children are attentive to each other and to the digital book (Tomasello et al., 2005).

The first excerpt shows the teacher relying on the children’s supposed knowledge of the Moomin universe, a strategy of activating prior knowledge from print book reading. As the children are not familiar with the literary universe, this strategy fails. In the second excerpt, she turns her attention toward the digital features of the app paratext presenting extratextual interactivity, and by this, exploiting the potential to activate prior knowledge within this specific pre-reading.

### Before Reading the Picture Book App *Yesper and Noper*

In video observation 612appJA4, the kindergarten teacher and four children^2^ (one boy and three girls) prepare to read the picture book app *Yesper and Noper* (Stai, 2008). The setup is the same as in the first reading session, and the teacher implements the “show strategy” (Hoel and Tønnessen, 2019). In preparation for the shared reading, the teacher has taken advantage of recording opportunities in the app, and she has created her own soundtrack for the book. The discussion starts with the screensaver image on the iPad: a picture of the teacher, who has dressed up as a professor, and the children argue over whether it is the teacher. Then, they talk about their number tags, and they start adding numbers. “Have you started school yet?” the teacher teases. The children have read the print version of the book earlier.

### Paratext in the Picture Book App *Yesper and Noper*

**FIGURE 4:**
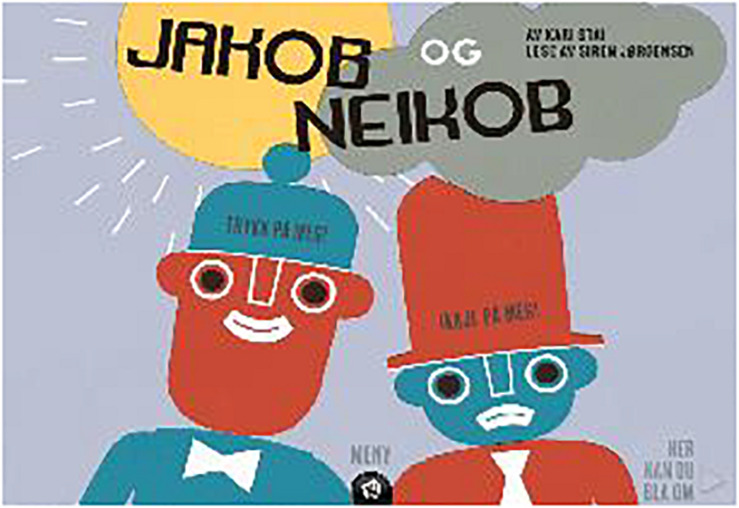
Opening page of the app *Yesper and Noper.* Screenshot reproduced with permissions from Kari Stai and Det Norske Samlaget.

In the app version of *Yesper and Noper*, a catchy melody with verbal text explaining who Yesper and Noper are starts immediately. As in the print version of the book, the opening page of the app displays the name of the author, the book title, and a full-page illustration of the characters Yesper and Noper (Figure 4). Interactivity is added to the illustration: On Yesper’s hat is written “Press me!” and on Noper’s hat “Not me!” When these hot spots are tapped, the characters say either “Yes” or “No.” The menu opens when the publisher’s logo at the bottom of the page is tapped. The choices in the menu are “Sound effects” (yes/no), “Show written text” (yes/no), “Read to me” (yes/own recording/no), and “Play the latest sound recording.” In the bottom right corner, it says, “Here you can turn the page.” Page 2 in the menu gives an overview of the pages in the app, instructions for recording and playing the reader’s own sound, and a memory game based on illustrations from the book.

As in the analysis of the first event, we looked at the dialog and interaction taking place prior to the reading activity.

### Establishing Joint Attention

The screensaver image on the tablet captures the children’s attention immediately, and to recruit (Wood et al., 1976) the children to the shared reading activity, the teacher uses the children’s knowledge of the book as a common starting point, saying: “This time I have a book that you have seen before [shows the screen], and what is it called?” “Yesper and Noper!,” the children reply. The teacher’s thorough planning, creating a soundtrack for the book, adds enthusiasm and helps to anchor the children’s attention and dedication.

T: We will do it like this. You can choose if you want to hear the voice that is on the iPad [touches the Yesper character and activates the soundtrack].
App: Press me [with the voice of the kindergarten teacher].
C (all): [Smile and laugh as their eyes move between the screen and the teacher].
C (5): He said “Press me”, “Press me”.
T: [touches the Noper character and activates the soundtrack]
App: Not me [with the voice of the kindergarten teacher].
C (5): “Not me”, “Not me” [mimics the voice in the app].
C (all): [Laugh].
T: Have you heard that voice before?
C (several): Yes/No.
C (6): It’s you!
T: Are you absolutely sure?
C (all): Yes [smiling].
C (6): Put that voice back on.

As in the reading of the Moomin app, the interactive and intratextual hot spots attract the children’s attention. They are eager to take turns tapping. Instead of making strict rules and denying the children access, the teacher allows the children to tap the screen one at a time, thus reducing the children’s degrees of freedom (Wood et al., 1976).

T: Would you like to try pressing it? [extends the tablet toward child 5].
C (5): [Taps on Yesper and smiles, then taps on Noper; the other children watch closely].
C (5): No [mimics the voice in the app].
C (5): I see both.
T: [Extends the tablet toward child 6].
C (6): [Taps quickly, first on Yesper, then on Noper].
C (5): [Laughs out loud].
T: [Extends the tablet toward child 4].
(4): [Taps repeatedly, and the publisher’s logo appears on the screen].
C (4): Hey.
T: Oi, what happened now? ‘Loading’, it says.
C (4): It was because I pressed so many times.
T: Do you think that’s why?
C (5): It was I who did it many times.
T: Yes, now we can read a completely blank book.
C (all): [Laugh and smile]

Even as the children take advantage of their opportunity to rapidly tap the hot spots and the app starts reloading, the teacher keeps calm and turns the situation into a joke where she reminds the children of the purpose of the picture book app, namely, reading.

Before the reading starts, the children also build their expectations for the digital affordances, which the new medium adds to the story. In this reading of *Yesper and Noper*, the new element is the teacher’s own soundtrack.

T: Should we listen to the voice on the iPad then?
C (all): Yes
T: There are two different voices on the iPad.
C (6): You!
T: Yes, and then there is another lady.
C (all): You, you [point at the teacher].
T: Would you like to hear how the second lady is first?
C (all): No, you.
C (6): You first.
C (all): You first.
T: OK.

This is an example of joint attention, where the children unite their interest and show that they greatly appreciate the teacher’s preparation.

Similar to in the Moomin reading, the pre-reading in this example starts before the children have seen the book. However, the paratext of the Yesper and Noper app differs from that of the Moomin app in that it invites readers to engage in the literary universe before the story starts also *via* intratextual interactivity (Zhao and Unsworth, 2017). One might say that pre-reading is implemented in the paratext of this app, still on the premises of the medium, with built-in hot spots and sound. The teacher actively uses these digital features to initiate a frame of joint attention.

### Activating Prior Knowledge

The teacher contributes to the children’s comprehension of the text by helping them activate their prior knowledge. The children have read the book earlier: they know the story and the characters. Nevertheless, by describing what they see in the paratext such as the opening illustration, they are reminded of the contrasts between the characters (colors, clothes, temper, and mood) and thus of the main plot of the story.

T: Yes, is there any other difference between them?
C (6): [Leaning forward and pointing] There is sun and rain.
C (4): [Leaning forward and pointing] And he’s angry, and he’s happy.
T: Yes.
C (2): [Stands up, takes a few steps toward the board and points] He has blue here, and he has red here.
T: Yes, it’s true.
C (6): And he has a hat, and he has cap.
T: Mmhmm [affirms].
C (5): And Yesper has sun, and Noper has… [looking for the word] rain!
T: Why do you think so?
C (2): And then Yesper has red ears, and then Noper has blue eyes, no, blue ears [laughs].
T: Mmhmm [affirms].
T: Why do you think there is a sun over Yesper and rain over Noper?
C (6) [pointing]: He’s glad, and he’s angry.
C (2): Angry [confirms].
C (5): [Makes an angry facial expression, clenches his hands and grins].

Even though the children know the story, they express their appreciation of this recall process. They focus on details in the illustrations, put forward hypotheses, and are active and co-creative based on their experience horizon (Rogoff, 1997). In this way, the children’s prior knowledge forms the basis for a deeper understanding of the text. In the children’s investigation of the details in the illustration, they are attentive, and they use verbal as well as body language to communicate observations and interpretations of observations.

The strategy to activate prior knowledge bears a resemblance to the pre-reading phase in traditional print book readings. The children establish and widen the literary universe by elaborating on the contrasting illustrations of Yesper and Noper, driven by questions as well as confirmations from the teacher. This activation of prior knowledge is directly linked to the characters and the text. In this case, the teacher’s questions do not activate prior knowledge related to the medium and the digital affordances within the paratext, for example, the soundtrack and the intratextual hot spots: “Why does Yesper say ‘Yes’ and Noper say ‘No’?” This might be seen as a missed opportunity.

## Conclusion and Implications for Practice

In this study, we sought to explore what happens in the minutes before shared reading starts by asking: How much time is spent on establishing joint attention and activating prior knowledge before reading print picture books and picture book apps? In addition: How does establishing joint attention and activating prior knowledge manifest in two examples of app readings characterized by the longest pre-reading phase durations in the sample?

Our results show that there is great variation in kindergarten teachers’ practice with regard to the time spent on pre-reading, and based on qualitative inspection, it is slightly more common with short pre-reading phases for picture book app readings than for print book readings (Figure 1 and Table 2). The wide-ranging variation between the different readings is clearly an expression of the different teachers’ pre-reading strategies; however, held together by the benefits that pre-reading provides (Bråten, 2007; Roe, 2008), it is also an expression of great differences in children’s opportunities for language learning and text comprehension. This finding implies a need for knowledge in the field of practice in regard to providing equal learning opportunities for all children in kindergarten.

Short time spent on pre-reading in story times with app readings might indicate that the app medium does not invite joint attention and activation of prior knowledge to the same degree as do print books. However, the results might indicate that we need to look for new ways to invite joint attention and activation of prior knowledge starting with the possibilities that the medium provides. The two examples that we present in this article, which might be considered best-practice examples due to the strategies employed by the teacher, highlight the great potential for pre-reading found within the paratext of the picture book apps. In these examples, traditional and unorthodox methods are employed.

In the two pre-reading recordings, we found clear manifestations of activating prior knowledge and joint attention. The design of the paratext differs between each story; however, within the relatively short time before reading starts, it seems to promote teacher behavior that is associated with establishing joint attention. The menus are important in creating and forming the children’s expectations on the shared reading and the picture book app. In the Moomin app, these instructions and extratextual interactivity are not part of the narrative’s meaning-making, yet they play an important part in what happens prior to the reading activity because the teacher recognizes this potential and insists on making room for elaborating the digital affordances. In the Yesper and Noper app, the reading instruction “Play own sound” adds to the frame of joint attention and expectations of the text.

In our examples, the teacher goes beyond the picture book apps and exploits the potential of the medium itself for joint attention and activation of prior knowledge. The digital bookshelf and even the screen saver represent possibilities. Thus, the digital medium provides a unique potential to start pre-reading in new ways, unknown from print book reading. To recognize this potential affords the teacher a frame for establishing joint attention and activating prior knowledge, even though the medium does not invite this specific action.

With regard to the activation of prior knowledge, the teacher’s strategies are traditional, especially in the example of Yesper and Noper, where the paratext serves as an invitation to highlight the illustrations of the main characters both to recruit the children’s joint attention and to activate prior knowledge. In the Moomin app, the children guess and speculate, yet this strategy fails because they have no prior knowledge of the Moomin universe. The teacher’s new and creative strategy is provided by the paratext: the menu giving instructions on how to read the app. Even if the menu might be considered to be an instruction for the reader physically holding the device, this teacher chooses to use this as an opening for activating prior knowledge and connecting new and familiar elements within the digital medium. Finding creative ways to exploit the possibilities of the paratext might mark the difference between the teachers who spend time on pre-reading in app readings and those who do not.

## Study Limitations

Based on our material, we find clear examples of pre-reading strategies specific to app readings. As the sample is small, more research is needed to deepen knowledge of pre-reading in ECEC institutions in general and with reference to digital books specifically. In our article, we have focused on best-practice examples of pre-reading events. Nevertheless, a knowledge gap remains concerning app readings with short or no pre-reading phases. As digital books present new ways of reading, our study contributes to addressing the great need for knowledge on how to promote joint attention and activation of prior knowledge in these new ways of reading.

## Data Availability Statement

The qualitative data and transcripts of the specific video data that support the conclusions of this article will be made available by the authors, without undue reservation.

## Ethics Statement

The studies involving human participants were reviewed and approved by the Norwegian Centre for Research Data. Written informed consent to participate in this study was provided by the participants’ legal guardian/next of kin.

## Author Contributions

TH participated in design of the overall study, collecting and coding of data. TH and ES conceived the original idea for this manuscript. ES and KS-H performed the quantitative analysis. TH and ES analyzed the qualitative data and interpreted results. TH and ES wrote the manuscript. All authors contributed to the article and approved the submitted version.

## Conflict of Interest

The authors declare that the research was conducted in the absence of any commercial or financial relationships that could be construed as a potential conflict of interest.

## Footnotes

1 ECEC institutions for children aged 1–5 years are called kindergartens in Norway.

2 The children with number tags 1 and 3 are not present during this reading event.

## References

[B1] Aisato L. (2014). *En fisk til Luna.* Oslo: Gyldendal.

[B2] Arnott L. (2018). Children’s negotiation tactics and socio-emotional self-regulation in child-led play experiences: the influence of the preschool pedagogic culture. *Early Child Dev. Care* 188 951–965. 10.1080/03004430.2018.1443919

[B3] Bae B. (2004). *Dialoger Mellom Førskolelærer Og Barn - En Beskrivende og Fortolkende Studie.* UiO: Avhandling til graden Dr. Philos, Det utdanningsvitenskapelige fakultetet, Institutt for spesialpedagogikk.

[B4] Bishop D. V. M. (2014). *Uncommon Understanding: Development and Disorders of Language Comprehension in Children.* London: Psychology Press.

[B5] Bråten I. (ed.) (2007). *Leseforståelse: Lesing I Kunnskapssamfunnet-Teori Og Praksis.* Oslo: Cappelen akademisk forlag.

[B6] Bråthen C. Markhus R. (2013). *Frøet.* Oslo: Gyldendal.

[B7] Burger K. (2015). Effective early childhood care and education: successful approaches and didactic strategies for fostering child development. *Eur. Early Childhood Educ. Res. J.* 23 743–760. 10.1080/1350293x.2014.882076

[B8] Carpenter M. Nagell K. Tomasello M. Butterworth G. Moore C. (1998). Social cognition, joint attention, and communicative competence from 9 to 15 months of age. *Monogr. Soc. Res. Child Dev.* 63 1–174. 10.1515/9783110802047-fm 9835078

[B9] Cresswell J. W. (2007). *Qualitative Inquiry & Research Design (Vol. 2).* California: Sage Publications.

[B10] Dickinson D. K. Morse A. B. (2019). *Connecting Through Talk. Nurturing Children’s Development with Language.* Baltimore: Brookes.

[B11] Genette G. (1997). *Paratexts: Thresholds of Interpretation*, Vol. 20 Cambridge, MA: Cambridge University Press.

[B12] Grøver V. (2018). *Å lære språk i barnehagen [Learning language in kindergarten].* Oslo: Cappelen Damm akademiske.

[B13] Hoel T. Wagner Å. K. H. Oxborough G. H. O. (2011). Lesefrø: språkstimulering gjennom leseaktiviteter i barnehagen. [Language stimulation through reading activities in kindergarten]. *Cappelen Damm Akademisk.*

[B14] Hoel T. Tønnessen E. S. (2019). Organizing shared digital reading in groups: optimizing the affordances of text and medium. *AERA Open* 5 1–14. 10.1177/2332858419883822

[B15] Hoel T. Jernes M. (2020). Samtalebasert lesing av bildebok-apper: barnehagelærer versus hotspoter [Dialogue based reading of picture book apps: kindergarten teacher versus hotspots]. *Norsk Pedagogisk Tidsskrift* 104 121–133. 10.18261/issn.1504-2987-2020-02-04

[B16] Jansson T. (2017). *Hvordan gikk det? (orig. Hur gick det sen?).* Oslo: Cappelen Damm.

[B17] Keene E. O. Zimmermann S. (1997). *Mosaic of Thought: Teaching Comprehension in a Reader’s Workshop.* Oslo: Cappelen Damm akademiske.

[B18] MacWhinney Brian (1991). *The Childes Project: Tools for Analyzing Talk.* Oslo: MacWhinney Brian.

[B19] Mangen A. Hoel T. Jernes M. Moser T. (2019). Shared, dialogue-based reading with books vs tablets in early childhood education and care: protocol for a mixed-methods intervention study. *Int. J. Educ. Res.* 97 88–98. 10.1016/j.ijer.2019.07.002

[B20] Mangold (2010). *INTERACT quicstart manual V2.4. Online.* Arnstorf: Mangold International GmbH.

[B21] Merchant G. (2015). Keep taking the tablets: iPads, story apps and early literacy. *Austral. J. Lang. Literacy* 38 3–11.

[B22] Mol S. E. Bus A. G. (2011). To read or not to read: a meta-analysis of print exposure from infancy to early adulthood. *Psychol. Bull.* 137 267–296. 10.1037/a0021890 21219054

[B23] Mol S. E. Bus A. G. de Jong M. T. (2009). Interactive book reading in early education: a tool to stimulate print knowledge as well as oral language. *Rev. Educ. Res.* 79 979–1007. 10.3102/0034654309332561

[B24] Mol S. E. Bus A. G. de Jong M. T. Smeets D. J. H. (2008). Added value of dialogic parent–child book readings: a meta-analysis. *Early Educ. Dev.* 19 7–26. 10.1080/10409280701838603

[B25] Pesco D. Gagné A. (2017). Scaffolding narrative skills: a meta-analysis of instruction in early childhood settings. *Early Educ. Dev.* 28 773–793. 10.1080/10409289.2015.1060800

[B26] Riessman C. K. (2008). *Narrative Methods for the Human Sciences.* Los Angeles: Sage Publications.

[B27] Roe A. (2008). *Lesedidaktikk: etter den første leseopplæringen.* Oslo: Universitetsforlaget.

[B28] Rogoff B. (1997). Evaluating development in the process of participation: theory, methods, and practice building on each other. *Change Dev.* 13 265–285.

[B29] Roskos K. Burstein K. You B.-K. (2012). A typology for observing children’s engagement with eBooks at preschool. *J. Interact. Online Learn.* 11 47–66.

[B30] Schwebs T. (2014). Affordances of an app. A reading of the fantastic flying books of mr. morris lessmore. *Nordic J. ChildLit Aesth.* 5:24169 10.3402/blft.v5.24169

[B31] Stai K. (2008). *Jakob & Neikob.* Oslo: Samlaget.

[B32] Tomasello M. (2003). *Constructing a Language. A Usage-Based Theory of Language Acquisition.* Cambridge, MA: Harvard university press.

[B33] Tomasello M. Carpenter M. Call J. Behne T. Moll H. (2005). Understanding and sharing intentions: the origins of cultural cognition. *Behav. Brain Sci.* 28 675–691. 10.1017/s0140525x05000129 16262930

[B34] Tønnessen E. S. (2016). “Bildebokresepsjon på skjerm; nye erfaringer av det litterære,” in *Litteratur inter artes. Nordisk litteratur i samspill med andre kunstarter*, eds Langas U. Sanders K. (Kristiansand: Portal forlag), 345–366.

[B35] van den Broek P. Kendeou P. Lousberg S. Visser G. (2017). Preparing for reading comprehension: fostering text comprehension skills in preschool and early elementary school children. *Int. Electron. J. Element. Educ.* 4 259–268.

[B36] Wood D. Bruner J. Ross G. (1976). The role of tutoring in problemsolving. *J. Child Psychol. Psychiatry* 17 89–100. 932126 10.1111/j.1469-7610.1976.tb00381.x

[B37] Zhao S. Unsworth L. (2017). “Touch design and narrative interpretation: a social semiotic approach to picture book apps,” in *Apps, Technology and Younger Learners*, eds Kucirckova N. Falloon G. (New York: Routledge), 89–101.

